# Impact Behavior and Residual Strength of PEEK/CF-Laminated Composites with Various Stacking Sequences

**DOI:** 10.3390/polym16050717

**Published:** 2024-03-06

**Authors:** Alexander V. Eremin, Mikhail V. Burkov, Alexey A. Bogdanov, Anastasia A. Kononova, Pavel S. Lyubutin

**Affiliations:** Laboratory of Mechanics of Polymer Composite Materials, Institute of Strength Physics and Materials Science of Siberian Branch of Russian Academy of Sciences, 634055 Tomsk, Russia; burkovispms@mail.ru (M.V.B.); ispmsbogdanov@gmail.com (A.A.B.); kononova_aa@ispms.ru (A.A.K.); p.lyubutin@gmail.com (P.S.L.)

**Keywords:** polymer composite, polyetheretherketone, carbon fiber, stacking sequence, mechanical properties, impact behavior, finite element modelling

## Abstract

Carbon fiber-reinforced composites are popular due to their high strength and light weight; thus, the structures demonstrate high performance and specific strength. However, these composites are susceptible to impact damage. The objective of this research was to study the behavior of carbon fiber-reinforced laminates based on a polyetheretherketone (PEEK) matrix with six stacking sequences under static and impact loading. Four-point bending, short-beam bending, drop weight impact, and compression after impact tests were carried out. The results were complemented with digital shearography to estimate the damaged areas. Finite element modeling served to assess the failure mechanisms, such as fiber and matrix failure, in different layers due to tension of compression. Three behavior pattern of layups under drop-weight impact were found: (i)—energy redistribution due to mostly linear behavior (like a trampoline) and thus lower kinetic energy absorption for damage initiation, (ii)—moderate absorption of energy with initiation and propagation of concentrated damage with depressed redistribution of energy in the material, (iii)—moderate energy absorption with good redistribution due to initiation of small, dispersed damage. The results can be used to predict the mechanical behavior of composites with different stacking sequences in materials for proper structural design.

## 1. Introduction

In structural design, not only the properties in static loads but also the dynamic behavior of the material plays great importance. Polymer fiber reinforced laminates, which are especially used in sports equipment, cars, or aircrafts, must be resistant to impacts and maintain high residual strength in the presence of damage. One of the widespread high-strength composites is epoxy reinforced with carbon fibers (CFs). It has excellent specific strength; however, its fracture toughness is quite low, making it prone to impact damaging and delamination. One of the approaches to enhance the material is to additionally reinforce the matrix using dispersed fillers [1]. This increases mechanical properties, particularly interlaminar and impact toughness. Another way is to make a three-dimensional composite by stitching the layers of fabric perpendicular to the plane of the laminate [2]. To solve the problem of low impact resistance, one may look for other matrix materials or reinforcing fibers. For example, implementing aramid fibers instead of carbon ones or using tougher thermoplastic matrices versus more brittle thermosets.

Polyetheretherketone (PEEK) is a promising thermoplastic polymer with high strength, low density, and ability to operate at elevated temperatures. A number of advantages of PEEK over epoxy resin have been demonstrated in the past [3,4]. Thus, residual compressive strength after impact for carbon-fiber reinforced laminates is significantly greater for PEEK than epoxy because delamination is less extensive. PEEK attracts quite a lot of attention from researchers. A study of its properties is being carried out for both states: unreinforced and reinforced with fibers. Mechanical properties were studied under various types of static loading as well as under impact. For example, Hu et al. [5] studied mechanical responses and damage behaviors of PEEK composites reinforced with carbon fiber fabric that were subjected to low-velocity impacts (LVIs).

An option to increase impact strength is to add additional layers such as polyetherimide (PEI) films between CF/PEEK layers [6]. The addition of PEI film was found to improve Mode I and Mode II interlaminar fracture toughness. For low-energy impacts (10 J), PEI-reinforced panels were found to perform well. However, the improvement of properties minimized as the impact energy increased. Compression after impact (CAI) and combined load compression (CLC) tests showed that although the PEI inserts reduced the laminate intact compressive strength, they exhibited higher relative residual strength than other laminates tested.

Another approach for improving the properties of laminated composites is to vary the stacking sequence. Strait et al. [7] conducted impact tests to characterize carbon fiber-reinforced polymer (CFRP) laminates. No significant effect of layup was found in terms of the energy required to cause damage. However, the maximum load is highly dependent on the stacking sequence. Replacing the woven fabric with unidirectional tape in a quasi-isotropic layup resulted in a significant reduction in energy to maximum load. The authors conclude that stacking sequence and shape of the reinforcement can have significant impact on toughness, especially at high impact energies.

Alhayek et al. [8] studied the effects of different layups of glass fibers on the flexural strength of pultruded polymers. At four-point bending, the layups—(±45/0/90/0/90/0) and (±45/90/0/±45)—showed a flexural strength of 399.9 MPa and 242.5 MPa, respectively.

The effects of layups on the properties of the composites was also studied using numerical calculations by Pradhan et al. [9]. It was observed that the available stiffness of a laminate with a crack was largely dependent on orientation angle of the outer layers, number of cracked cross-layers, and number of unbroken outer layers ±θ in the laminate.

It is obvious that layup affects bending and impact strength, since the distance of a certain layer from the middle plane makes a different contribution resulting in a non-uniform distribution of stresses and strains throughout the thickness of the laminate. 

A number of investigations have been carried out by scientific teams varying CF/PEEK laminate layups in addition to those already mentioned earlier [3,4]. In a series of works by Morita et al. [10,11,12,13], several layups of carbon fiber-reinforced PEEK, namely (0/+30/0/−30)_S_, (0/+60/0/−60)_S_, and (0/+45/90/−45)_S_, have been studied. It was found that the damage area depends linearly on the impact energy. Moreover, the ratio of the damaged area to the impact energy is smaller the less the difference in the angles of rotation of the fibers in neighboring layers.

Liu et al. [14] compared the effects of two different stacking sequences ([0°/90°]_8S_ and [0 °/45°/90°/−45°]_4S_) at four impact energies. The peak impact force with an increase in impact energies ranged from 7.8 kN/8.3 kN to 11.4 kN/13.7 kN, and the CAI strength ranged from 370.5 MPa/419.3 MPa to 212.8 MPa/232.5 MPa. At the same time, the relative change in residual strength at impact energies of 0, 15, and 30 J for the second layup is minimal, while for the first it ranges up to 30%. At higher impact energies (45 and 60 J), the relative drop in strength becomes approximately the same.

The paper by Wang et al. [15] presents the investigation on the properties of CF/PEEK laminates with cross-ply layups: A [0_5_/90_5_/0_5_], B [0_3_/90_3_/0_3_/90_3_/0_3_] and C [0/90]_15_. In A and B laminates, transverse cracks occurred at very low impact energies (~0.5–1 J). Delamination initiated at approximately 3 J and fiber rupture began to occur after 10 J. Laminate C exhibited very limited transverse cracking and delamination until penetration occurred at 13 J.

Simulations can be an important part of understanding the fracture processes of composites. In addition, a correctly designed model makes it possible to predict the behavior of a material under new conditions or with changed properties of the material itself. To model layered composite materials, the finite element method is often used in modern research. Layers are specified individually, considering the anisotropy of the properties of each layer. The interaction of layers can specified using the cohesive behavior of the contact planes without increasing the number of nodes or elements [14].

The important point in the simulation is the selection of fracture criterion for matrix and fibers or a composite as a whole. One of the possible criterion in the literature is the Puck criterion [16]. The Puck failure theory is a stress-based criterion applicable for unidirectional composite lamina developed for both matrix and fiber failure modes. In [14], Liu et al. used the Puck criterion to model matrix failure under compression. Numerical simulation errors for peak impact force, impact energy absorption, and CAI strength were 3.8–14.8%, 3.7–6.9%, and 2.2–6.7%, respectively.

Probably, the most popular way for simulate failure is the implementation of the Hashin criterion [17]. Liu et al. [18] describes a detailed experimental and numerical study of the behavior of composite laminates under a low-velocity impact with energy of 15 J and different geometry of the impactor. A three-dimensional finite element (FE) model with the damage initiation mechanisms defined in the Hashin damage criteria, which was implemented as a subroutine for Abaqus/Explicit, was employed to simulate the impact event and to investigate the effects of the impactor’s geometry. The numerical predictions, including those for the loading response and the damage maps, provided good agreement with the experimental results. Thus, for a hemispherical steel impactor, the maximum load, displacement, and area damage for experimental studies were 4.7 kN, 6.0 mm, and 1180 mm^2^, while for simulation, they amounted to 4.5 kN, 5.9 mm, and 1090 mm^2^, respectively. The same parameters for a flat impactor for the experiment are 7.1 kN, 4.7 mm, 1060 mm^2^, and 7.0 kN, 4.6 mm, and 980 mm^2^ for the simulation.

Ouyang et al. [19] simulated low-velocity impact and compression after the impact experiments of two composite materials at different energy levels with the Hashin criterion and virtual crack-closure technique, respectively. The predicted failure modes and CAI strengths of laminates are in good agreement with the experiment results from this paper and the literature at various impact energies, which proves the validity of the model. The predicted compression strengths after different impact energies of laminates had the errors relative to the average experiment values of 2.2%, 8.4%, 1.3%, 0.5%, 3.4%, and 3.6%, depending on testing conditions. For the studied M21 epoxy resin laminates in the impact energy range from 6.5 J to 29.5 J, the errors between the experimental and numerical results of CAI strength were 4.3%, 12.3%, 6.5%, and 5.3%.

Based on the results of the literature review, it can be seen that a sufficient number of works, including ones from recent years, deal with the influence of layups on the impact properties of laminated composites. However, most of them consider layups that do not meet the requirements described in [20] or the number of layups in the study is limited to two or three types.

The paper is aimed to estimate the effects of six different layups of PEEK/CF composites on their mechanical properties during static and impact tests. Furthermore, this work is directed to evaluate layups with higher properties and reveal the mechanics of the failure process.

## 2. Materials and Methods

### 2.1. Manufacturing Procedure

Toray TC1200 PEEK prepregs were chosen for laminate preparation. The layers of prepregs were stacked in the required layup and molded using hot pressing. The laminates had a rectangular shape of 215 × 150 mm, and the specimens for testing were cut out using CNC milling machine with polycrystalline diamond mill.

For the selection of the layups in the experiment, the basic rules and restrictions for the design and production of composites provided by Ntourmas et al. [20]:

1. Symmetry. In order to avoid coupling between bending and extension symmetric laminates are usually preferred.
2. Balance. Balanced laminates can be used to remove coupling between shear and extension. Balanced laminates are formed by an equal number of layers with +θ and −θ orientations (θ ≠ 0°, 90°).
3. Damage tolerance. Plies placed on the external part of the laminate should not be in the direction of the principal load path. In most cases, a layer oriented at +45° or −45° is placed on the outer part of the laminate.
4. Minimum percentage. In favor of minimizing matrix degradation and encouraging a fiber-dominated failure mode, a minimum percentage of all fiber orientations might be desirable in a laminate. The minimum percentage rule makes sense for laminates which use the four standard fiber orientations (0, 90, 45, −45). This design rule is commonly used in conjunction with a maximum allowed percentage.
5. Grouping. In order to decrease the coupling between bending and twist, +θ and −θ layers may be grouped together.
6. Contiguity. According to the contiguity design rule, the maximum number of consecutive layers placed in the same orientation is limited. This is carried out in order to minimize interlaminar stresses and encourage a more uniform stress distribution.
7. Disorientation. To minimize interlaminar shear effects, the absolute difference between the fiber orientations of adjacent laminae might be limited to a maximum of 45°.

Although all the proposed rules may be desirable in the design process, they cannot be applied simultaneously as they conflict with each other and may lead to an unsolvable optimization problem. 

The molded and tested layups were symmetrical, balanced, and quasi-isotropic. Some of the rules presented here (above) were violated in order to assess the changes in mechanical behavior. There were six types of stacking sequences, as follows:

1. [−45/0/45/90]_4S_—quasi-isotropic layup proposed in [21,22] for testing of impact toughness of fiber reinforced laminates. The layup has a 45°-oriented fiber layer on the surface and 45° interface between any layers.
2. [0/−45/90/45]_4S_—the layup with layers swapped in each pair (−45/0 and 45/90), resulting in a layup with 0°-oriented surface layer and 45° interface.
3. [−45/45/0/90]_4S_—the layup with 0 and 45 swapped, resulting in an interface of 90° between layers in −45/45 and 0/90 pairs. The interface between these pairs is 45°.
4. [0/90/45/−45]_4S_—the same as layup #3, but the −45/45 and 0/90 pairs are swapped. Thus, the surface layer is 0°-oriented.
5. [(−45/45)_2_/(0/90)_2_]_2S_—the same as #3, but each layer pair is repeated two times. The 90° interface is between the layers in (−45/45)_2_ and (0/90)_2_ groups and 90° between groups. In the resulting layup, −45/45 layers are located closer to the surface while 0/90 s are shifted towards midplane.
6. [(0/90)_2_/(−45/45)_2_]_2S_—the same as layup #5, but the (−45/45)_2_ and (0/90)_2_ groups are swapped.

### 2.2. Mechanical Tests

There were four mechanical tests performed to characterize the effects of layup on mechanical behavior. Flexural properties of laminates were assessed according to four-point bending procedure B of [23]. The rate of crosshead movement was 1 mm/min. The specimen thickness was ~4.4 mm, and the span was set to 88 mm, resulting in a span-to-thickness ratio of 20:1.

Stress at the outer surface in the midpoint of span and strain in bending test can be calculated using the following equations:

*σ* = 3*PL*/(4*bh*^2^); *ε* = 4.36*δh*/*L*^2^; δ = 11/8 *d_l_*,

σ—stress at the outer surface at mid-span, MPa;
P—applied force, N;
L—support span, mm;
b—width of beam, mm;
h—thickness of beam, mm;
ε—maximum strain at the outer surface, mm/mm (or × 100%);
δ—mid-span deflection, mm;
*d_l_*—displacement of the load points of movable grip, mm.

The second testing was performed according to [24] to determine short-beam strength (SBS), which may be related to interlaminar shear strength. SBS is less affected by the layup than flexural strength, and these properties together can be used to characterize CFRPs. The test is included herein mostly for quality control. 

Specimen sizes are calculated relative to thickness *t*, which is ~4.4 mm. Width is 2 × *t*, while span is 4 × *t*. SBS is calculated as follows:

*F^SBS^* = 0.75 *P_m_*/*bh*,

*F^SBS^*—short-beam strength, MPa;
*P_m_*—maximum load observed during the test, N;
*b*—measured specimen width, mm;
*h*—measured specimen thickness, mm.

The third and fourth tests are jointly performed. The impact properties of the laminate might be divided into two parts: impact damaging via drop weight test and resistance to impact damage tested under compression of the impacted laminate.

The composite laminate is subjected to a drop-weight impact event according to [21], and then [22] determines the residual compressive strength after impact. Kinetic energy of the impact was 30 J. According to the thickness of 4.4 mm, the specific energy was 6.7 J/mm.

The specimens for testing are rectangular shaped with the dimensions of 100 × 150 mm. The cylindrical impactor with a hemispherical nose had a weight of 5.4 kg and a diameter of 16 mm. The specimens are clamped in a fixture with a window of 75 × 125 mm. The impactor falls down to the center of the plate with a velocity prior to collision of ~3.3 m/s. The impact with the level of kinetic energy used in the test results in the formation of damage in the CFRP. 

**Figure 1:**
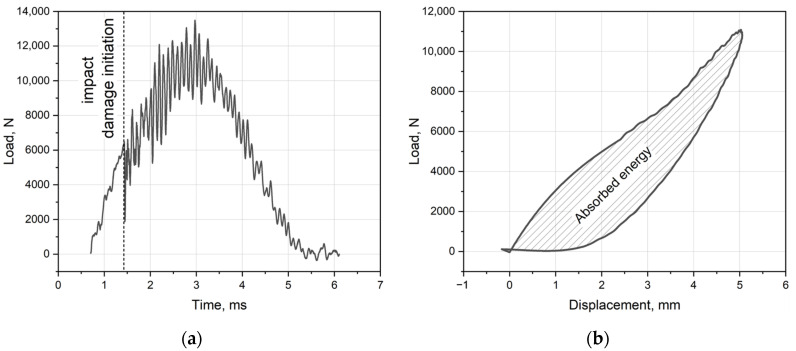
Results obtained after impact test: (**a**) “Load–time” diagram with impact damage initiation. (**b**) “Load–displacement” diagram and the area for calculation of absorbed energy.

Behavior of composites under impact loading might be estimated using several parameters:

- Peak load—maximum force obtained in the loading diagram (Figure 1a);
- Damage load—force when the load drops in the diagram, indicating the origin of damage (dotted line in Figure 1a);
- Damage energy—energy lost by the impactor and transferred to the specimen at the moment of damage origin;
- Absorbed energy—calculated as an area delineated by a “load–displacement” diagram, as illustrated in Figure 1b. Absorbed energy also might be calculated as a difference between kinetic energy of impactor before and after impact. However, it includes energy loss via friction, heating, etc. Energy obtained from hysteresis area is slightly less than difference of kinetic energies.

### 2.3. Inspection Using Digital Shearography

Digital shearography was used to estimate the damage size after impact event. The technique implements a very sensitive optical analysis of a deformation pattern on the specimen’s surface. The surface was illuminated with a 532 nm (green) Nd:YAG laser to generate a speckle pattern. The specimen was heated with an IR lamp and during cooling; the photos of the speckle pattern for shearography were obtained. 

During cooling, thermal flows provide deformation gradients in the composite, which also changes the laser speckle pattern on the surface. So, the post processing of speckle pattern images could derive the strain pattern on the specimen surface. When the plate is free of damage, the strain pattern is homogenous; however, in presence of any damage, it becomes irregular. The larger the damage, the larger the inhomogeneity observed in the processed image. 

### 2.4. Finite Element Modelling of Impact Behavior

A full-scale simulation was carried out using ABAQUS 2019/Explicit. Material properties were obtained from the manufacturer’s datasheet and with preliminary experiments. Longitudinal and transverse Young’s moduli were E_11_ = 135 GPa and E_22_ = E_33_ = 10 GPa; shear moduli were G_12_ = G_13_ = 5.2 GPa and G_23_ = 3.8 GPa. Poisson ratios were υ_12_ = υ_13_ = 0.301 and υ_23_ = 0.396. Tensile and compressive strengths in the fiber direction were X_t_ = 2410 MPa and X_c_ = 1300 MPa; in the transverse direction, they were Y_t_ = 86 MPa and Y_c_ = 189 MPa, respectively. Longitudinal and transverse shear strengths were S_12_ = S_13_ = 152 MPa and S_23_ = 81 MPa. Material density was ρ = 1600 kg/m^3^.

The Hashin criterion [17] was used to simulate elements’ failure, including four different damage initiation mechanisms. Hashin criteria comprehensively consider various damage modes, such as fiber failure in tension, matrix cracking due to transverse tension and shear, fiber compressive damage (buckling or kinking) and matrix crushing under transverse compression, and shear effects. The Hashin theory is based on the separation of different failure modes and has been proven to be effective for the study of the onset and the evolution of fiber and matrix failures [25,26,27]. The Hashin criterion works quite correctly when assessing the fracture of fibers, but is not very effective in the failure of the matrix, especially during compression [14]. 

Failure energies for the Hashin criterion are as follows: longitudinal tensile and compressive fracture energy G_XT_ = 90,000 J/m^2^ and G_XC_ = 60,000 J/m^2^; transverse tensile and compressive fracture energy G_YT_ = 1500 J/m^2^ and G_YC_ = 5000 J/m^2^. This criterion was chosen for models because it allows us to determine the most critical damage process after impact and was already implemented in Abaqus Explicit. 

The laminate was modeled as deformable solid. The interaction between layers was described by cohesive behavior with the following parameters of damage initiation, propagation, and stabilization. Fracture energies were G_IC_ = 1.6 kJ/m^2^ and G_IIC_ = 2.3 kJ/m^2^, linear degradation law based on the mixed mode law of the Benzeggagh–Kenane criterion was equal to 1.5, and the viscosity coefficient was 0.007. The Benzeggagh–Kenane criterion was used as the fracture criterion [28], which demonstrates high efficiency for composite materials with the use of identical critical fracture energies along the two shear directions. The Benzeggagh–Kenane fracture criterion is particularly useful when the critical fracture energies during deformation, purely along the first and the second shear directions, are the same. 

All laminate layers were modeled using SC8R continuum shell elements. Metal impactors were modeled as 3D rigid (discretely rigid) elements. The interaction between a composite laminate and impactor was defined as a rigid contact with a friction coefficient of 0.3.

The boundary conditions reproduce real test conditions—the rectangular plate was positioned in the rectangular frame and pressed with supports. The weight, shape, and size of the impactor complied with the [21]. The impactor motion was determined by the initial velocity, measured in experiment before it struck the specimen.

## 3. Results

### 3.1. Flexural Tests and Short-Beam Strength

**Figure 2:**
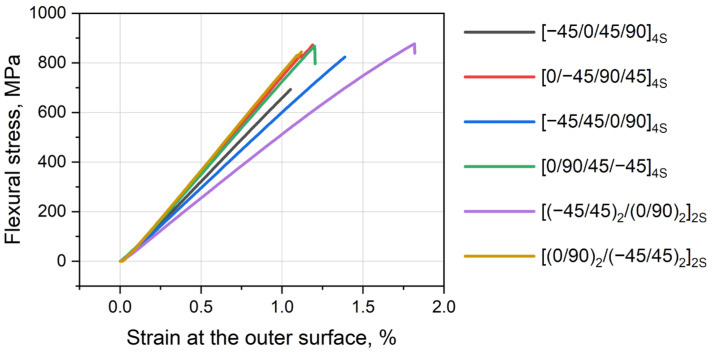
Stress–strain diagram of flexural tests of PEEK composites with various layups.

The diagrams of the calculated strain on the outer surface vs. flexural stress were plotted in Figure 2 for [23] tests. Table 1 presents the results on mechanical properties obtained according to [24]: strength, strain at failure, flexural modulus, and short-beam strength.

The diagram demonstrates that most stacking sequences have linear behavior under bending loads and have abrupt failure. Nonlinearity is visually observed for [(−45/45)_2_/(0/90)_2_]_2S_. According to Figure 2 and Table 1, various layups result in various flexural moduli and strengths. The highest flexural moduli were observed for layups where 0 layers were on the outer surfaces of the laminate ([(0/90)_2_/(−45/45)_2_]_2S_, [0/−45/90/45]_4S_ and [0/90/45/−45]_4S_), while 45/−45 layers on the outer surface of composite reduced flexural stiffness, e.g., [(−45/45)_2_/(0/90)_2_]_2S_. Moderate moduli were observed for [−45/0/45/90]_4S_ and [−45/45/0/90]_4S_. When fiber direction coincides with tension and compression forces (0° layer), it provides higher stiffness. The 45° layer had less longitudinal stiffness than 0° one. The 90° layer was even weaker than 45°. e.g., [0/90/45/−45]_4S_ had a slightly lower modulus than [0/−45/90/45]_4S_ (50.6 ± 1.2 vs. 53.1 ± 1.9 GPa). Thus, to obtain a higher flexural modulus, it is preferred to place 0° layers at the outer surface of the laminate.

However, high modulus does not guarantee high failure stress. The leaders in strength are [0/−45/90/45]_4S_ and [0/90/45/−45]_4S_, while [(0/90)_2_/(−45/45)_2_]_2S_ takes only 4th place, being adrift by approximately 65 MPa (or 7%). This layup is too stiff and thus has a lower ability to redistribute stresses, resulting in brittle failure. Surprisingly, the [(−45/45)_2_/(0/90)_2_]_2S_ layup, having ±45 fiber on the outer space and the lowest flexural modulus, demonstrated good flexural strength. It should be noted that in most cases, failure occurs at the inner surface due to fiber compression and buckling of the layer.

Short-beam strength is mostly related to the interlaminar properties under in-plane shear loading (close to mode II interlaminar fracture toughness). So, the results are nearly the same for almost all layups, showing sufficient adhesion between layers. It should be noted that a lower SBS was observed for layups where a 90 interface was in the midplane of the specimen (where shear stresses are the highest). 

### 3.2. Low-Velocity Impact and Compression after Impact

The testing machine is able to measure impactor displacement, velocity, and contact load in the impactor head. The key results measured in drop-weight tests are presented in Table 2, which include peak load, damage load, damage energy, and absorbed energy.

Kinetic energy absorbed by the laminate can be estimated via the difference in kinetic energy of an impactor at the beginning of impact and after rebound from a laminate surface. It should be considered that this difference includes the loss of energy due to inertia and the friction in guide bearings. So, it is better to directly derive the energy from the loading diagrams. 

**Figure 3:**
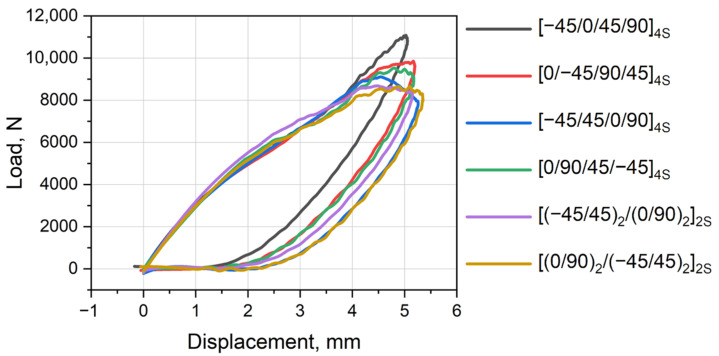
Load–displacement diagram (namely mechanical hysteresis loops) of impact tests of PEEK composites with various layups.

The typical smoothed load–displacement curves for each laminate layup can be obtained and are plotted in Figure 3. The diagram shows loading and unloading of a specimen; thus, the area delineated by the curve characterizes absorbed energy, which is spent mostly on damage nucleation and propagation. Of course, some minor portion of energy is dissipated on internal and contact friction or heating. The key parameters of the loading and absorbed energy (as curve delineated area) are presented in Table 2. In should be noted that a higher maximum load does not mean higher absorbed energy.

The subsequent step for testing the damaged laminate is compression after impact. This is performed to estimate residual strength, which greatly depends on damage size and interlaminar strength. The results are also presented in Table 2.

The lowest absorbed energy had specimen of a [−45/0/45/90]_4S_ layup, which resulted in the highest CAI strength and peak load. The composite demonstrated higher energy absorption by elastic straining and reduced the damage nucleation process. The energy for the first damage initiation was rather low—7.3 J (last but one); however, during further loading, this laminate was damaged less. It should be noted that the laminate at impact loading was clamped on the perimeter, which resulted in combined bending around two axes. Thus, it was difficult to easily use the flexural modulus and strength of the exact layup in comparison to the CAI strength. However, at CAI, the specimens were loaded along 0° axis via compression; thus, the layup should have determined the residual strength. It should be important what fiber layers are most damaged. For example, 0°-oriented plies dominate in resistance to compression, and their damage should probably decrease CAI strength. During the analysis of CAI for various layups, it was difficult to establish any regularity depending on the layup as the process of impact loading and subsequent compression is too complex in nature. The next section with the results of the shearography damage evaluation can assist with this. 

Nevertheless, some minor points can be noted. Two layups—[0/−45/90/45]_4S_ and [−45/45/0/90]_4S_—demonstrated high absorbed energies and thus a low CAI strength. The absorbed energy for [−45/0/45/90]_4S_ was the lowest one, resulting in the highest CAI strength. Both layups—[(−45/45)_2_/(0/90)_2_]_2S_ and [(0/90)_2_/(−45/45)_2_]_2S_—where the 0/90 and −45/45 layer pairs were grouped twice, demonstrated the lowest peak loads during impact and the highest damage energy, resulting in good residual CAI strength.

### 3.3. Digital Shearography

**Figure 4:**
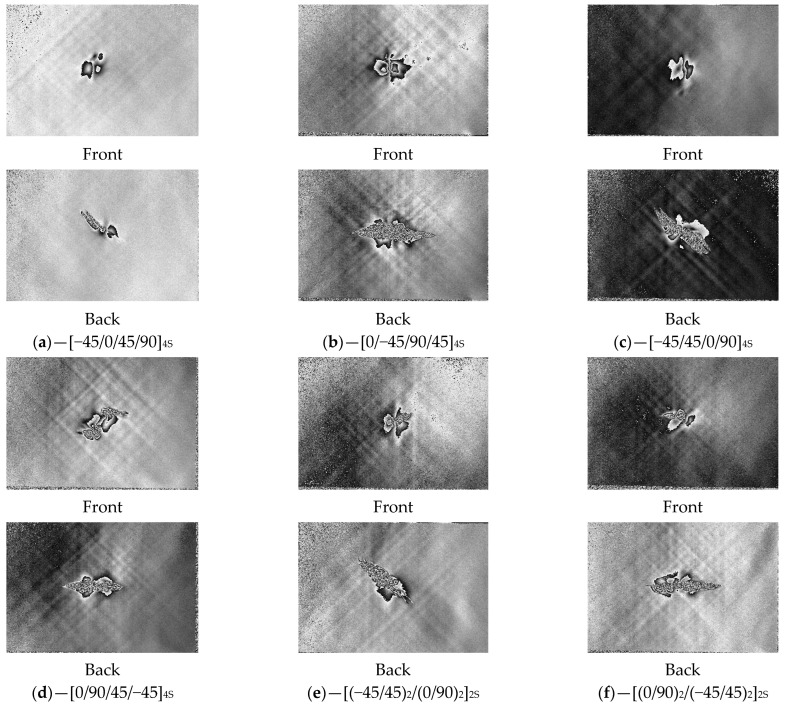
Front and back images of impacted PEEK composites with various layups obtained via digital shearography (**a**–**f**).

Figure 4 represents the examples of impact damage visualized using digital shearography, which are seen as inhomogeneities in the central parts of the photos. Images are presented with their front and back sides for each specimen type. The edges of the images correspond to the edges of the specimens, so the relative size of the damage might be compared to the total area of the laminate. 

Damage area estimated using digital shearography is presented in Table 3. It can be seen that smaller damage with a moderate scatter is in the front side of the impacted panel and almost twice-as-large damage with more significant scatter is on the back side. The results are consistent with the standard impact theory. This theory states that for thick laminates based on brittle fiber–matrix systems (like CFRPs), the cross-sectional damage profile looks like a “pine tree”. The damage in this case was caused by normal in-plane stress, which exceeded the transverse tensile stress. It initiated matrix failure at the back side, developing into delamination and fiber breakage. 

Due to the visual analysis, some general results might be drawn. The damage to the front surface was smaller than that at the back surface. The layup had a minor effect on the damage shape at the front, while at the back, the top layer delaminated more extensively and thus affected the shape of damage. The shape of damage at the back surface was elongated in the horizontal direction for layups with a 0°-orientate direction of fiber on the outer layer (Figure 4b,d,f). On the contrary, for layups with 90° at outer layers, the damage at the back surface was elongated in a diagonal direction: from upper left to bottom right.

It can be observed that area of damage on the back surface had a good correlation with CAI residual strength for nearly all layups. The impact damage in the laminate was mainly located in the half opposed to the impact surface, and it dominated the residual bearing capacity.

### 3.4. Damage Modelled via Finite Element Technique

**Figure 5:**
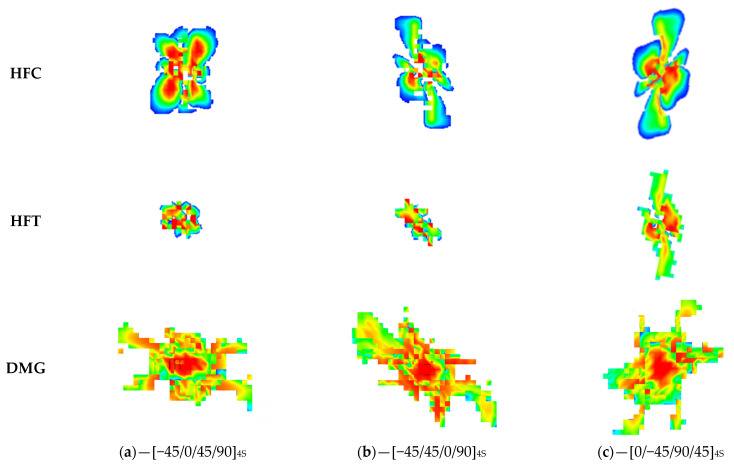

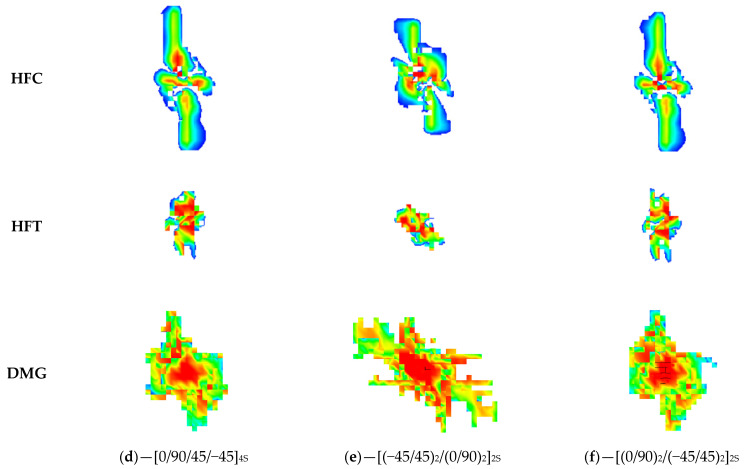
Damage in various layups according to different failure criteria. HFC—Hashin failure criterion for fiber compression, HFT—Hashin failure criterion for fiber tension, DMG—overall damage index for cohesive surfaces (denoted in ABAQUS as CSDMG). Blue-green-red color scale depicts damage index from 0.5 to 1.

Figure 5 demonstrates damaged elements in the laminate after impact obtained via an ABAQUS simulation. There are several failure criteria presented. HFC is the Hashin criterion for fibers that fail due to compression, and HFT is the Hashin criterion for the tension of fibers. The DMG criterion demonstrates overall failure of the cohesive surface (literally matrix failure and delamination). All these criteria take values from 0 to 1, where 0 represents the absence of damage and 1 represents a fully failed element. For visualization purposes, the Hashin and DMG criteria have an index equal to 0.5, so their images include failed and partially damaged elements.

Results show that large, elongated damage originates mostly from fiber compression along the shorter side of the panel. Tensile damage has a more uniform shape, depending on the direction, and also has a lower size than failure due to compression. Matrix (delamination) failure is mostly concentrated along the −45/45 directions.

In general, it might be noted that the presence of 0/90 layers at the front outer surface leads to dominant damage in the 90 direction, which is seen for the HFC criterion in Figure 5d,e when compared to the other layups. However, this layup reduces matrix failure, especially in the −45/45 directions (DMG criterion in Figure 5d,e). Compression failure in the 90° direction has a minor effect on compression properties in the 0 direction when the compression after impact test is performed because these broken fibers are perpendicular to the loading axis. However, a non-damaged matrix is beneficial for preventing the longitudinal delamination of layers.

## 4. Discussion on Layup Selection

After the results were obtained and analyzed, it was found that, similar to [7], maximum load is highly dependent on the stacking sequence with variations of nearly 20%. The peak load in this paper was in the range of 10.26 to 12.19 kN, resulting in a difference of 19%. Individual properties might have variations. The [(0/90)_2_/(−45/45)_2_]_2S_ layup has good compression strength after impact, high energy to initiate damage under impact, a significant flexural modulus, and moderate flexural strength. Peak load and absorbed energy are more complicated parameters which could not be directly evaluated. Not every higher value leads to a better performance. The [(0/90)_2_/(−45/45)_2_]_2S_ specimens have a low peak load (10.26 kN) and high residual strength (268 MPa), while the specimens with the [0/−45/90/45]_4S_ layup demonstrate a high peak load (11.80 kN) and low strength after impact (253.2 MPa). Moreover, [−45/0/45/90]_4S_ shows high strength (272.6 MPa) and peak load (12.19 kN) but the lowest absorbed energy (17.64 J). 

There are several ways how material may sustain an impact event:

A high peak load and low absorbed energy result in globally linear behavior, like a trampoline. Thus, the high flexural modulus is not required to reach a high peak load. Small damage energy might be attributed to early fracture initiation but without significant propagation. An example can be made of the [−45/0/45/90]_4S_ layup, which demonstrates good resistance to impact loading. Similar findings can be found in [10,11,12,13], supporting the finding that the damaged area of the laminate is smaller the lower the difference in the angles of rotation of the fibers is in the neighboring layers. The abovementioned layup has all the interfaces of 45 degrees between its adjacent layers, and its good impact resistance and residual strength were confirmed with tests. However, such a rule cannot be generally used in all instances, and some layups demonstrate high absorbed energy but small damage, resulting in high CAI strength. 

A high peak load and high absorbed energy with small damage energy result in the early initiation of damage, large damage propagation to absorb kinetic energy, and high stiffness to demonstrate a high peak load. However, this leads to small strength after impact. At the same time, some specimens demonstrate a low peak load, high absorbed energy, and low damage energy. The difference between low and high peak loads is the result of a difference in the flexural modulus. The lower the modulus, the lower the peak load. So, [0/−45/90/45]_4S_ and [−45/45/0/90]_4S_ demonstrate low resistance to impact loading.

Sufficiently high damage energy and good strength after impact accompanied with good CAI strength are connected with a later initiation of damage; thus, small damages are distributed on a large area for the absorption of kinetic energy. However, these damages should be small enough to have minor effects on deformation behavior. This redistribution of energy results in high strength. The [0/90/45/−45]_4S_, [(0/90)_2_/(−45/45)_2_]_2S_, and [(−45/45)_2_/(0/90)_2_]_2S_ layups demonstrate good resistance to impact and the difference in peak load attributed to the flexural modulus.

Mechanical properties are dependent on the layup, but their effects are not obvious. The selection of the optimal layup which possesses the best combination of impact and flexural properties might be challenging. The authors would like to propose a simple estimation for selecting them. Firstly, the most valuable properties were determined with a condition that a higher value equates to better properties. The list of properties chosen for comparison is as follows:

- Flexural strength, MPa;
- Flexural modulus, GPa;
- SBS, MPa;
- Damage energy, J;
- CAI strength, MPa.

**Figure 6:**
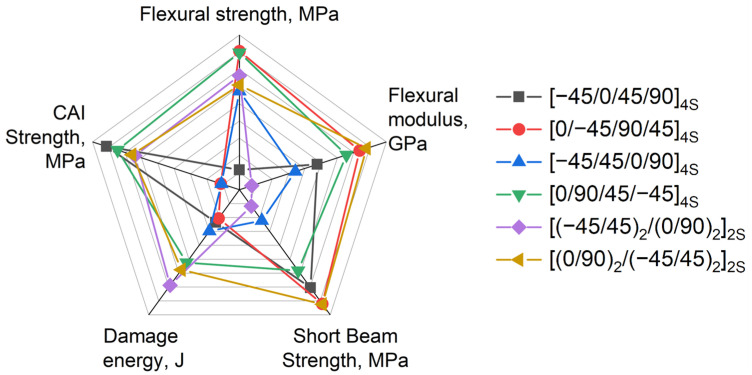
Normalized (divided by max) radar diagram of valuable mechanical properties for various layups.

The chosen properties for each layup were divided by the max to normalize values. The results are summarized in Table 4. The value which equals to 1.00 means that this layup has the highest value compared to other layups. A five-axis radar diagram was plotted based on normalized values (Figure 6). Each axis is representative of each mechanical property in the normalized values. The radar diagram may help with visually comparing all the stacking sequences.

The question of “what layup is better?” might be answered via an analysis of the area delineated by the diagram for each layup or simply by calculating the sum of the normalized values for each layup. As far as the diagram is normalized, the contribution of each property is equal and does not depend on an absolute value. The sums are presented in Table 4.

According to described procedure, the best layups according to the aforementioned properties are the [(0/90)_2_/(−45/45)_2_]_2S_ and [0/90/45/−45]_4S_ layups. These layups have very close normalized sums—4.85 and 4.82. The worst layups also have similar overall properties. The third place is taken by the [0/−45/90/45]_4S_ layup, which has a sum of 4.65 due to having significantly less damage energy. The fourth place is taken by the [(−45/45)_2_/(0/90)_2_]_2S_ layup, which has a sum of 4.55 and a reduced flexural modulus. The last two places go to the [−45/45/0/90]_4S_ and [−45/0/45/90]_4S_ layups, which have very similar summarized values—4.93 and 4.37. These layups showed decreased properties; however, the [−45/0/45/90]_4S_ layup demonstrated the highest CAI strength.

## 5. Conclusions

This manuscript presented the results of PEEK/CF composites that were impact tested with different layups. Preliminary flexural tests according to [23] (long beam) showed different strengths and flexural moduli for various layups. Stiffness was determined by fibers with the [0°] direction at the outer surface. Strength is not always associated with [0°] fibers only as fractures occur mostly due to compression at the inner surface. The short-beam strength test (according to [24]) showed nearly the same interlaminar properties for different layups, and showed sufficient adhesion between layers.

The effects of layups on the impact properties of composites were estimated according to [21] for low-velocity impacts and then in accordance with [22] for compression tests to determine their strengths after various impacts. The three ways of how the different layups may behave under and after impact are described in detail in the “Discussion” section: (i)—energy redistribution due to linear behavior (like a trampoline), which causes kinetic energy absorption for damage initiation, (ii)—moderate absorption of energy with initiation and propagation of concentrated damage with depressed redistribution of energy in the material, (iii)—moderate energy absorption with good redistribution due to initiation of small dispersed damage. 

Modelling results show the contribution of several failure mechanisms to the overall damage that occurs and their dominant behavior depending on the layup. Layers with 0/90 fibers at the front outer surface result in dominant compression damage in the 90° direction; moreover, they reduce matrix failure, especially in the −45/45 directions. 

To answer the question of what layup is better, five of the most valuable parameters were chosen—flexural strength, flexural modulus, short-beam strength, damage energy, and compression strength after impact. The higher the parameter value, the higher performance of the layup. In summary, the best layups in relation to the most of properties are the [(0/90)_2_/(−45/45)_2_]_2S_ and [0/90/45/−45]_4S_ layups.

## Figures and Tables

**Table 1:** Flexural properties of tested PEEK composites with various layups.

Layup	Flexural Strength, MPa	Failure Strain, %	Flexural Modulus, GPa	SBS, MPa
[−45/0/45/90]_4S_	698 ± 33	1.46 ± 0.05	47.2 ± 0.9	91.8 ± 0.6
[0/−45/90/45]_4S_	926 ± 71	1.76 ± 0.14	53.1 ± 1.9	93.1 ± 1.3
[−45/45/0/90]_4S_	849 ± 72	1.96 ± 0.16	44.5 ± 0.4	86.4 ± 1.6
[0/90/45/−45]_4S_	923 ± 53	1.94 ± 0.06	50.6 ± 1.2	90.4 ± 1.4
[(−45/45)_2_/(0/90)_2_]_2S_	879 ± 5	2.54 ± 0.04	37.5 ± 1.1	85.3 ± 1.5
[(0/90)_2_/(−45/45)_2_]_2S_	861 ± 27	1.58 ± 0.05	53.4 ± 0.5	93.1 ± 0.9

**Table 2:** Summary of the properties of PEEK composites with various layups for impact and compression after impact tests.

Layup	Peak Load, kN	Damage Load, kN	Damage Energy, J	Absorbed Energy, J	CAI Strength, MPa
[−45/0/45/90]_4S_	12.19 ± 1.25	6.25 ± 0.19	7.30 ± 0.41	17.64 ± 2.41	272.6 ± 20.1
[0/−45/90/45]_4S_	11.80 ± 0.14	6.12 ± 0.70	7.15 ± 1.27	20.88 ± 2.14	253.2 ± 3.1
[−45/45/0/90]_4S_	10.64 ± 0.61	6.45 ± 0.45	7.65 ± 0.75	21.87 ± 0.69	253.1 ± 14.7
[0/90/45/−45]_4S_	11.18 ± 0.37	6.92 ± 0.16	8.91 ± 1.05	20.20 ± 1.25	270.7 ± 5.8
[(−45/45)_2_/(0/90)_2_]_2S_	10.19 ± 0.16	7.34 ± 0.23	9.82 ± 0.87	20.77 ± 0.47	267.8 ± 13.4
[(0/90)_2_/(−45/45)_2_]_2S_	10.26 ± 0.21	7.12 ± 0.32	9.20 ± 1.30	21.12 ± 0.89	268.3 ± 20.8

**Table 3:** Damage area of PEEK composites with various layups estimated via digital shearography.

Layup	Damage Area in Front Side, mm^2^	Damage Area in Back Side, mm^2^	Average Damage Area, mm^2^
[−45/0/45/90]_4S_	1116 ± 322	1893 ± 993	1504 ± 786
[0/−45/90/45]_4S_	1240 ± 230	2639 ± 313	1939 ± 804
[−45/45/0/90]_4S_	1497 ± 91	2760 ± 943	2129 ± 915
[0/90/45/−45]_4S_	1784 ± 446	2191 ± 496	1988 ± 477
[(−45/45)_2_/(0/90)_2_]_2S_	1413 ± 258	2450 ± 403	1932 ± 644
[(0/90)_2_/(−45/45)_2_]_2S_	1039 ± 202	1991 ± 339	1515 ± 578

**Table 4:** Summary for valuable mechanical properties of composites with various layups.

Layup	Normalized Properties					Sum of Normalized Mechanical Properties
	Flexural Strength	Flexural Modulus	SBS	Damage Energy	CAI Strength	
[−45/0/45/90]_4S_	0.75	0.88	0.99	0.74	1.00	4.37
[0/−45/90/45]_4S_	1.00	0.99	1.00	0.73	0.93	4.65
[−45/45/0/90]_4S_	0.92	0.83	0.93	0.78	0.93	4.39
[0/90/45/−45]_4S_	1.00	0.95	0.97	0.91	0.99	4.82
[(−45/45)_2_/(0/90)_2_]_2S_	0.95	0.70	0.92	1.00	0.98	4.55
[(0/90)_2_/(−45/45)_2_]_2S_	0.93	1.00	1.00	0.94	0.98	4.85

## Data Availability

Data are contained within this article.
